# Mould Routine Identification in the Clinical Laboratory by Matrix-Assisted Laser Desorption Ionization Time-Of-Flight Mass Spectrometry

**DOI:** 10.1371/journal.pone.0028425

**Published:** 2011-12-14

**Authors:** Carole Cassagne, Stéphane Ranque, Anne-Cécile Normand, Patrick Fourquet, Sandrine Thiebault, Chantal Planard, Marijke Hendrickx, Renaud Piarroux

**Affiliations:** 1 Laboratoire de Parasitologie-Mycologie, CHU Timone, Université de la Méditerranée, Marseille, France; 2 Service Protéomique, Centre d'Immunologie de Marseille Luminy, Marseille, France; 3 IHEM: Scientific Institute of Public Health, Mycology and Aerobiology Section, Brussels, Belgium; The Research Institute for Children, United States of America

## Abstract

**Background:**

MALDI-TOF MS recently emerged as a valuable identification tool for bacteria and yeasts and revolutionized the daily clinical laboratory routine. But it has not been established for routine mould identification. This study aimed to validate a standardized procedure for MALDI-TOF MS-based mould identification in clinical laboratory.

**Materials and Methods:**

First, pre-extraction and extraction procedures were optimized. With this standardized procedure, a 143 mould strains reference spectra library was built. Then, the mould isolates cultured from sequential clinical samples were prospectively subjected to this MALDI-TOF MS based-identification assay. MALDI-TOF MS-based identification was considered correct if it was concordant with the phenotypic identification; otherwise, the gold standard was DNA sequence comparison-based identification.

**Results:**

The optimized procedure comprised a culture on sabouraud-gentamicin-chloramphenicol agar followed by a chemical extraction of the fungal colonies with formic acid and acetonitril. The identification was done using a reference database built with references from at least four culture replicates. For five months, 197 clinical isolates were analyzed; 20 were excluded because they were not identified at the species level. MALDI-TOF MS-based approach correctly identified 87% (154/177) of the isolates analyzed in a routine clinical laboratory activity. It failed in 12% (21/177), whose species were not represented in the reference library. MALDI-TOF MS-based identification was correct in 154 out of the remaining 156 isolates. One *Beauveria bassiana* was not identified and one *Rhizopus oryzae* was misidentified as *Mucor circinelloides*.

**Conclusions:**

This work's seminal finding is that a standardized procedure can also be used for MALDI-TOF MS-based identification of a wide array of clinically relevant mould species. It thus makes it possible to identify moulds in the routine clinical laboratory setting and opens new avenues for the development of an integrated MALDI-TOF MS-based solution for the identification of any clinically relevant microorganism.

## Introduction

Fast and reliable identification of moulds would help manage the growing number of invasive mould infections, a leading cause of morbidity and lethality in immunocompromised patients [1]. Currently, mould identification relies on the macroscopic and microscopic observation of colonies grown on mycological media. Adequate phenotypic identification of moulds requires highly skilled mycologists, who are found in a few reference laboratories. Moreover, some species phenotypically indistinguishable have been described based on DNA sequence analysis of rRNA or other protein-coding genes. Yet, even DNA sequence-based identification of moulds has several limitations. The DNA extraction yield may be relatively low because mould cells are hard to lyse. PCR amplification may fail due to the presence of PCR inhibitors in mould cultures. Moreover, although it may technically succeed, the molecular identification of moulds would require at least 5 to 7 days in the routine clinical laboratory setting. This delay negatively impacts the patients' prognosis [2]. Finally, only some clinical laboratories routinely use a molecular approach for microorganism identification [3]. In 2007, only 17% of the US clinical laboratories performed molecular analysis [4]. Therefore the identification of moulds remains problematic and misidentifications likely occur in the routine setting [5].

A novel microorganism identification method has emerged in bacteriology that is based on MALDI-TOF mass spectrum (MS) analysis. This MALDI-TOF MS-based identification technique analyzes the protein content from treated or intact cells of microorganisms under the form of a spectrum that is considered as a protein fingerprint specific of a micro-organism. An unknown microorganism is identified by comparing its spectrum with the spectra in the reference library [6]. MALDI-TOF MS-based identification is simple, fast, and accurate and has a high throughput for most bacteria. Numerous bacteriologists found that its identification accuracy outperformed that of conventional methods in the routine clinical laboratory setting [7]. A few preliminary studies aimed to identify moulds using MALDI-TOF MS [8]. However, each used only specific mould genera and culture conditions. Different extraction methods, types of matrix, and instruments were also used. This heterogeneity is particularly detrimental because mass spectra are influenced by culture conditions, extraction procedures, the type of matrix, and the spectrometer used [9]. Our study therefore sought to elaborate a standardized procedure suitable for the MALDI-TOF MS-based identification of clinically relevant moulds in the routine laboratory setting. In the first step, the operating procedures for MALDI-TOF MS-based identification were optimized and validated on a large panel of clinically relevant moulds. In the second step, we evaluated the performances of this MALDI-TOF MS-based approach for the identification of mould clinical isolates prospectively collected from the routine activity of the Marseille teaching hospital laboratory. Moulds were considered regardless of their phylogeny and relation to any specific clinical situation.

## Materials and Methods

### Fungal strains

Three panels of mould strains were used in this study. The panel P1 included 8 strains. The panel P2 included the 8 strains of the panel P1 and 11 other strains. The panel P3 (146 strains) included the strains of the panels P1 and P2 and 127 other strains. Panels 1 and 2 (Table S1) were used to optimize spectra acquisition. Panel 3 was used to validate the optimized process with clinical isolates. Of the 146 strains, 85 and 24 were graciously provided by respectively the BCCM/IHEM and the Pasteur Institute; they had been identified by macroscopic and microscopic examination by skilled mycologists and by ITS gene sequencing [10]. Beta-tubuline sequencing [11] was performed when required. The 37 remaining strains were recovered from clinical or environmental samples received at our lab (Table S1).

### Phenotypic and genotypic identifications

The clinical and environmental strains were first identified by skilled mycologists according to their phenotypic (macroscopic and microscopic) features following the keys of the Atlas of clinical fungi [12]. Identification was then confirmed by DNA sequence-based identification using both ITS1-5.8S-ITS2 and the D1-D2 variable region of the 28S unit of the rRNA gene as described by deHoog et al [13]. DNA extraction was performed using the QIAmp DNA kit (QIAGEN, France) and identified reaction sequences were run with a 3130 Genetic Analyzer (Applied Biosystem, Inc., Courtaboeuf, France). Resulting sequences were compared using the Medical fungi pairwise sequence alignment tool (http://www.cbs.knaw.nl/Medical/BioloMICSSequences.aspx).

### Fungal material extraction

The fungi were extracted using formic acid (FA) as follows. Fungal culture samples were mixed with 300 µL of sterile water (Water HPLC, Prolabo BDH, Fontenay-sous-Bois, France) and 900 µL of anhydrous ethyl alcohol (Carlo Erba SDS, Val de Reuil, France) in a sterile 1.5 ml tube. After 10 min centrifugation at 13,000 g, the pellet was incubated 5 min in 10 µL of 70% formic acid (Sigma-Aldrich, Lyon, France). Then 10 µL of 100% acetonitrile (Prolabo BDH) was added for 10 min of incubation before centrifugation (13,000 g, 2 min) and the supernatant was removed. Five pre-extraction operating procedures were tested.

- Fungi were cultivated on Sabouraud gentamicin-chloramphenicol agar plates (AES, France) and incubated 72 h at 27°C. The samples, i.e. fungal hyphae and spores, were harvested by gently scraping the fungal colonies with a sterile plastic device and then subjected to FA extraction as described above.
- The fungi were cultured for 24 h at 27°C on Sabouraud broth (BioMerieux, France). Fungal material was collected after 10 min centrifugation at 13,000 g. Then the pellet was washed three times with 1 ml of sterile water and suspended with 300 µL of HPLC sterile water and 900 µL of anhydrous ethyl alcohol in a sterile 1.5 ml tube subjected to FA extraction as described above.
- This procedure was identical to procedure A except that the hydro-alcoholic suspension of fungal material was heated 1 h at 95°C before extraction as described above.
- This procedure was identical to A except that the hydro-alcoholic suspension of fungal material was mechanically lysed by 3 cycles of micro-beads (Glass beads, acid-washed G4649 -≤106 µm, Sigma-Aldrich, Saint Quentin Fallavier, France) beating (60 seconds at power 6.5) with a FastPrep™-24 Instrument (MP Biomedicals, France) before extraction as described above.
- This procedure was identical to D except that the hydro-alcoholic suspension of fungal material was heated 1 h at 95°C before mechanical lysis as described above.

### Mass spectra acquisition and MALDI-TOF MS-based identification

A drop of 1 µL of supernatant was deposited on a spot of a polished steel target (MTP384 polished steel target, Bruker Daltonics GmbH, Bremen, Germany) and air-dried. Each spot was then covered with 1 µL of the matrix solution. The matrix solution was a daily-prepared saturated solution of α-cyano-4-hydroxycinnamic acid (HCCA) in 50% acetonitrile (and 2.5% trifluoroacetic acid (TFA) (Sigma-Aldrich, Lyon, France). The spectra were acquired after 650 shots in linear mode with an Autoflex speed™ I, Autoflex speed™ II, or UltrafleXtreme™ instrument (BrukerDaltonics, Germany) in the ion-positive mode with a 337 nm nitrogen laser. The following adjustments were used: delay: 170 ns; ion source1 voltage: 20 kV; ion source2 voltage: 18.5 kV; mass range: 3–20 kDa. The data were automatically acquired by the AutoXecute of the FlexControl v2.4 software and exported into MaldiBiotyper v2.1 software. Only peaks with a signal/noise ratio ≥10 were considered.

Reference spectra (MSP) were created by combining the results of 10 raw spectra per fungal isolate using the “MSP creation” function of the MaldiBiotyper software. LogScore values (LS) were obtained by comparing unknown sample spectra with a reference library using the “Start identification” function of the MaldiBiotyper software. LS-based identification results were considered correct when they agreed with those of phenotypic/genotypic identification.

Consecutive clinical isolates, cultured on Sabouraud gentamicin-chloramphenicol agar plates at 27°C for 72 h, were prospectively collected from the routine activity of the medical mycology laboratory at the teaching hospital of Marseille. They were identified using phenotypic criteria as described above. In parallel, samples were prepared according to procedure A, as described above. All isolates were spotted in quadruplicate and identified by MALDI-TOF MS using the largest library, which included the reference spectra of 146 filamentous fungi strains. The MS-comparison was based on the best-match LS values, as described in the reproducibility analysis paragraph.

The MS identification results of the clinical isolates were interpreted as follows:

If less than 3 out of the 4 spots issued from an isolate matched MSPs of the same species, the result of the MALDI-TOF MS-based identification for the isolate were considered non interpretable. This intra-spot discordance may be caused by a possible technical error. The clinical isolate was cultured, extracted, and submitted to MS identification again.
If at least 3 out of the 4 spots issued from a clinical isolate matched MSPs of the same species, the MALDI-TOF MS-based identifications were considered concordant and interpretable. If the MALDI-TOF MS-based identification matched the phenotypic one, the clinical isolate was considered correctly identified by MALDI-TOF MS. Otherwise the isolates were further identified by DNA sequence comparison as described above. If the identification by DNA sequence comparison gave the same result as the MALDI-TOF MS-based identification, the clinical isolate was considered correctly identified by MALDI-TOF MS, regardless of the phenotypic identification. If the DNA sequence comparison identification did not match the MALDI-TOF MS-based identification, the isolate was considered misidentified by MALDI-TOF MS.

### Comparison of extraction procedures

Five MSPs libraries (one for each pre-extraction operating procedure) were built. Each library contained eight MSPs, one for each of the eight first panel's isolates (P1). Each of these MSPs was derived from ten raw spectra. Each of the ten raw spectra composing an MSP was submitted to MS identification, and the best-match LS values were calculated using MaldiBiotyper. We then compared the number of peaks composing the spectra and the best-match LS values obtained with each strain of the panel with the five procedures. The C and E procedures were discarded at this step because they produced poor quality spectra.

To assess the technical reproducibility of the spectra obtained by procedures A, B, and D, we built three larger libraries, DB1-A, DB1-B, and DB1-D, which each included 19 MSPs. The MSPs were derived from 10 raw spectra obtained from a single culture of each strain included in the second panel (P2). Then, for each procedure, we compared each 190 raw spectrum to the 19 MSPs in the corresponding library and calculated the resulting best-match LS values. To test biological reproducibility, the 19 strains of panel P2 were further subcultured and extracted according to A, B, or D procedure. Then four spots of each extracted samples were compared to the MSPs in the corresponding library and LS values were analyzed.

### Statistical analysis

All statistical analyses were performed using two-sided tests and a p<0.05 significance level with the MASS package of the R software (http://www.r-project.org). The results of each extraction protocol were compared using the non-parametric rank sum Kruskal-Wallis test. In the case of a significant difference with the Kruskal-Wallis test, a post-hoc analysis was conducted using pairwise matched Wilcoxon tests with Holm's adjustment for multiple comparisons. The McNemar test without continuity correction was used for the pairwise comparisons of correct identification proportions.

### Reference spectra (MSP) library validation

A large library of reference spectra was constructed using extraction procedure A. This library included four MSPs (each of them derived from 10 raw spectra of four culture replicates) of 146 strains included in the P3 panel (Table S1). To assess the validity of this library, the 146 strains were sub-cultured again and submitted to extraction following pre-extraction procedure A. Four spots of each extracted sample were submitted to MS identification using this 146-reference spectra library, and the LS values of each spot were recorded.

## Results

### Comparison of extraction procedures

**Figure 1:**
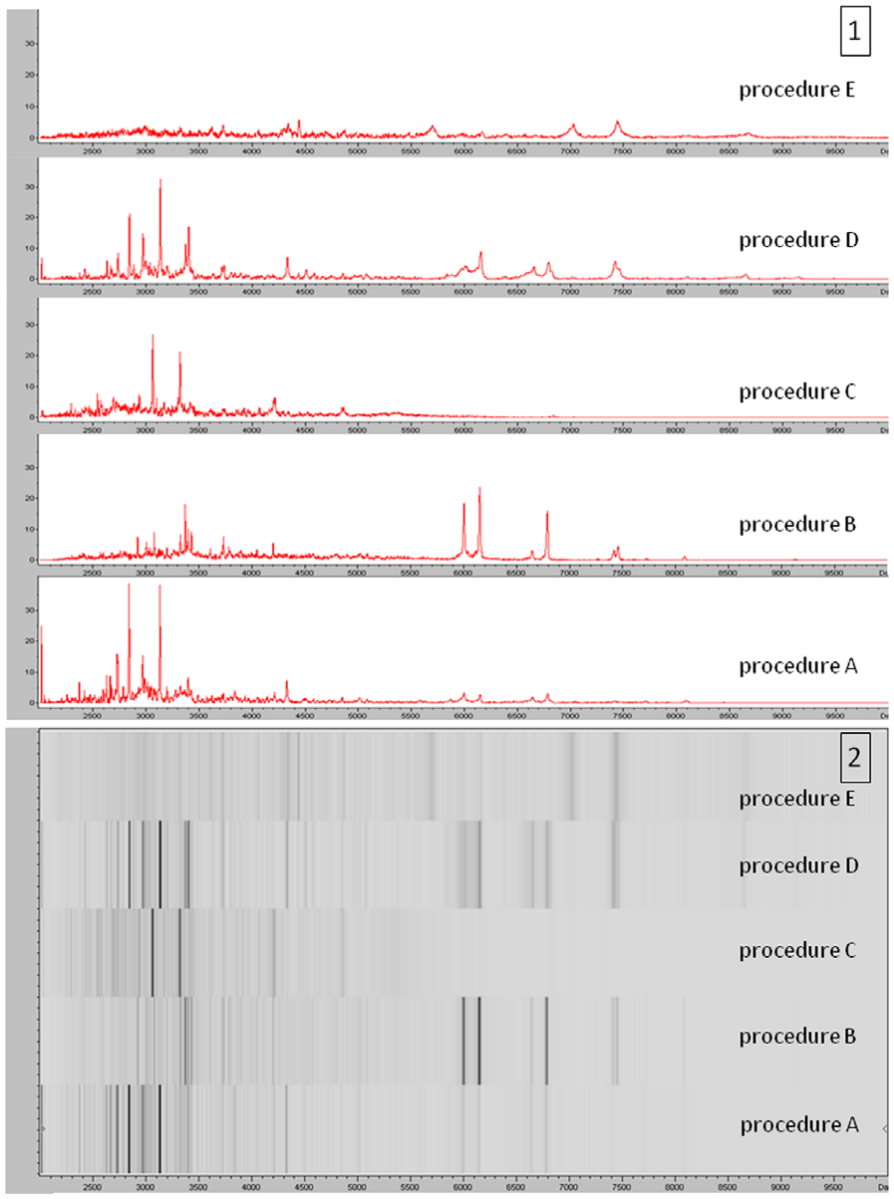
MALDI TOF MS spectra obtained with the 5 extraction procedures. (A) Gel view of the spectra of the *Aspergillus fumigatus* AFUM001strain obtained with the different extraction procedures. (B) List view of the spectra of the AFUM001obtained with the different extraction procedures. These procedures are detailed in the Material and Method section. The peaks obtained with procedure B, C and E were fewer and of lower intensity than those obtained with procedures A.

**Figure 2:**
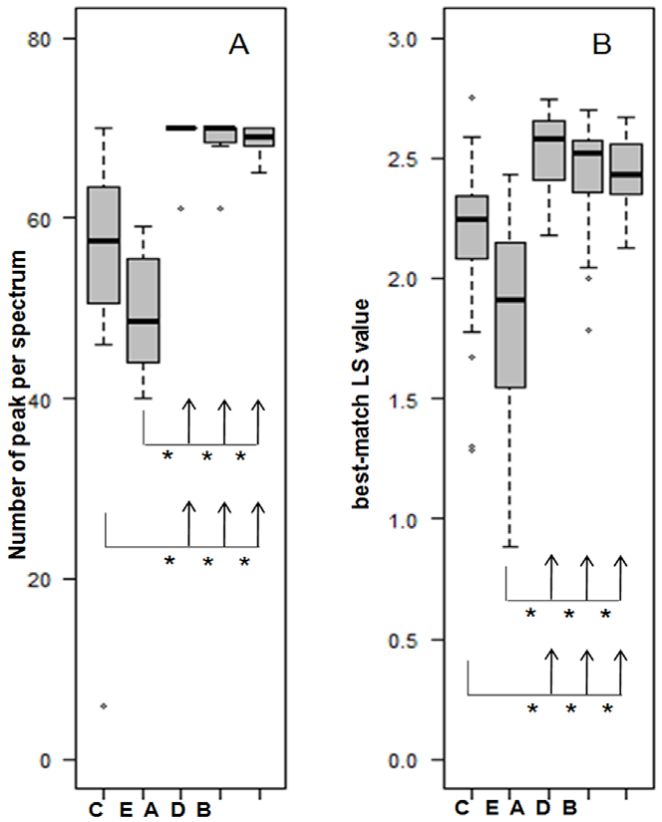
Comparison of the quality of spectra obtained from the 5 different extraction procedures. (A) Box-and-whisker diagrams of the number of peaks per spectra of the 5 procedures. (B) Box-and-whisker diagrams of the best-match LS value of the 5 procedures. The bottom and top of the box are the lower and upper quartiles, respectively; the band near the middle of the box represents the median; and the ends of the whiskers represent the lowest datum still within 1.5 interquartile range (IQR) of the lower quartile and the highest datum still within 1.5 IQR of the upper quartile. Asterisks indicate which pairs of procedures were statistically different (Wilcoxon tests, p<10^−3^). Procedures A, B, and D displayed significantly more peaks (p<10^−4^) and higher best-match LS values (p<10^−3^) than procedures C and E. No statistically significantly difference was found between procedure C and E on the one han and between procedures A, B, D on the other hand.

**Figure 3:**
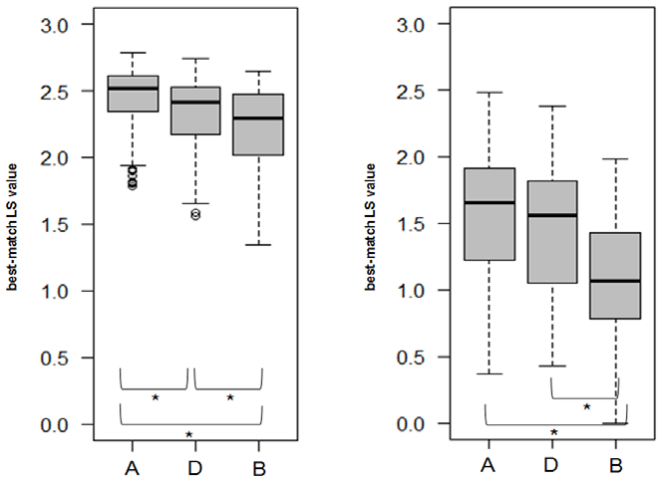
Comparison of the technical and biological reproducibility between the procedure A, B and D. (A) Box-and-whisker diagram of the best-match LS value obtained in the technical reproducibility test. (B) Box-and-whisker diagram of the best-match LS value obtained in the biological reproducibility test. The bottom and top of the box are the lower and upper quartiles, respectively; the dark band is the median; and the ends of the whiskers represent the lowest datum included in the 1.5 interquartile range (IQR) of the lower quartile and the highest datum included in the 1.5 IQR of the upper quartile). Asterisks indicate which pairs of procedures were statistically different (Wilcoxon tests, p<10^−3^). The pairwise matched Wilcoxon tests with Holm's adjustment for multiple comparisons indicated that the technical reproducibility was significantly better with procedure A than with procedures B and D (p<10^−16^ and p<10^−12^ respectively). Furthermore, the biological reproducibility with procedure B was significantly poorer than with procedure A (p<10^−9^).

The results with the 8-strains panel P1 indicated that both the number of peaks and the best-match LS values differed significantly with the procedures used (Kruskal Wallis tests for global heterogeneity: p<10^−15^ and p<10^−11^ respectively). The post-hoc analysis indicated that, on the one hand, procedures C and E, and, on the other hand, procedures A, B and D were not statistically significantly different (p>0.05). Procedures A, B, and D displayed significantly more peaks (p<10^−4^; Figure 1) and higher best-match LS values (p<10^−3^, Figure 2) than procedures C and E. Using the same statistical analysis with the 19-strains panel P2, we found that the best-match LS values for both technical and biological reproducibility assays differed between the procedures A, B and D (global heterogeneity tests, p<10^−15^ and p<10^−11^, respectively). Post-hoc analysis indicated that the technical reproducibility was significantly better with procedure A than with procedures B and D (p<10^−16^ and p<10^−12^ respectively). Furthermore, procedure B was rejected because its biological reproducibility was significantly poorer than procedure A (p<10^−9^, Figure 3). Finally, procedure D was rejected because it was more laborious than A. The ultimate library and further experiments were thus done following procedure A.

### MSP library validation

**Figure 4:**
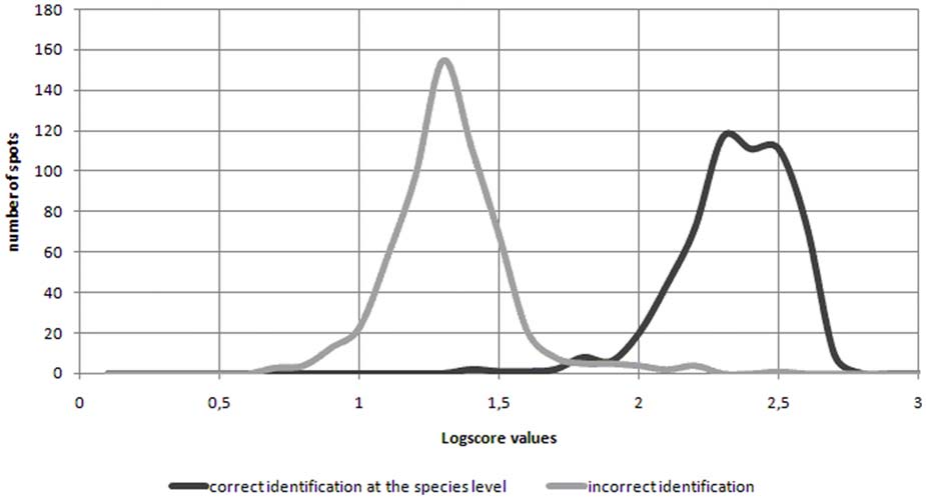
Distribution of the best-match LS values recorded during the mass spectra library validation test. The figure shows the best-match LS values for each of the 4 spots issued from a subculture of the 146 strains included in the library. The dark line represents the best-match LS values of the correct identification results whereas the gray line shows the best-match LS values of the misidentification results. The distributions of the best-match LS values for the spectra resulting either in a correct identification or a misidentification did almost not overlap.

The ultimate 146-strain library was first tested by comparing the raw MSPs of a subculture of each strain used to build the library with the MSPs in the library. All 584 spots tested (4 per strain) led to correct identification at the species level with high best-match LS (mean LS = 2.394+/−0.198). In contrast, for each spot, the maximum best-match LS score value corresponding to a misidentification (i.e. a species different from the one corresponding to the sample) was much lower (mean = 1.484+/−0.3104). Therefore, the distributions of the best-match LS values for the spectra resulting either in a correct identification or a misidentification did almost not overlap (Figure 4).

### Prospective MALDI-TOF MS-based identification of clinical isolates

From the 197 clinical isolates collected between July and November 2010, 177 (90%) could be correctly identified at the species level (i.e. because phenotypic and MALDI-TOF MS-based identifications were concordant, or by DNA sequence comparison-based identification) (Table 1). Twenty isolates were excluded from the evaluation because they were not identified to the species by DNA sequence comparison-based identification. The 177 identified isolates belonged to 33 species (Table 1). Twenty one isolates belonged to 16 species that were not represented by an MSP in our ultimate library and thus could not be identified. As expected, results of MS identification of these 21 isolates yielded low best-match LS value (mean = 1.369±0.176). Noticeably, intra-spot identification was discordant in each of these 21 isolates and no MALDI-TOF MS-based identification could be obtained. These results are important because they demonstrate that isolates whose species is not present in the reference library were not misidentified. The 156 remaining clinical isolates had at least one corresponding MSP from an isolate of the same species in the 146-strain library and could thus be correctly identified by our MALDI-TOF MS-based identification assay.

**Table 1:** Results of the polyphasic identification of 177 clinical mould isolates.

Species	nb of isolates	nb of concordancesmicro/spectro	nb of confirmationsby molecular biology	nb of strains of thespecies in the spectral bank
***Aspergillus fumigatus***	86	84	2	11
***Aspergillus niger***	12	11	1	4
***Alternaria tenuissima/alternata***	10	4	6	8
***Aspergillus flavus***	9	9	0	5
***Aspergillus terreus***	9	9	0	7
***Penicillium chrysogenum***	8	2	6	6
***Scedosporium apiospermum***	7	2	5	6
***Penicillium glabrum***	4	0	4	0
***Rhizopus oryzae***	3	0	3	4
***Aspergillus alliaceus***	2	0	2	0
***Aspergillus nidulans***	2	2	0	5
***Fusarium oxysporum***	2	1	1	5
***Geotrichum geomyces***	2	0	2	0
***Penicillium spirulosum***	2	0	2	1
***Aspergillus clavatus***	1	0	1	0
***Aspergillus melleus***	1	0	1	0
***Aspergillus oryzae***	1	0	1	0
***Aspergillus sydowi***	1	0	1	1
***Beauveria bassiana***	1	0	1	6
***Chaetomium globosum***	1	0	1	1
***Fomitopsis ostreiformis***	1	0	1	0
***Geosmithia pallida***	1	0	1	0
***Hamigera avallanea***	1	0	1	0
***Hypocrea jecorina***	1	0	1	0
***Nectria mauriticaula***	1	0	1	0
***Penicillium citrinum***	1	0	1	0
***Penicillium funiculosum***	1	0	1	0
***Penicillium oxalicum***	1	0	1	0
***Penicillium roqueforti***	1	0	1	1
***Penicillium spinophilum***	1	0	1	0
***Paecilomyces variotii***	1	0	1	4
***Trichoderma atroviride***	1	0	1	0
***Trichoderma viride***	1	1	0	2
**Total**	177	125	52	

(Nb, number; micro, phenotypic identification; spectro, MALDI TOF MS-based identification).

**Figure 5:**
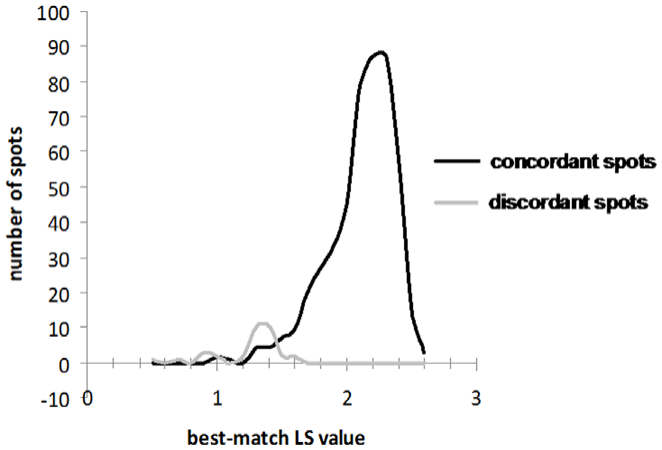
Distribution of the best-match LS values issued from the identification of the clinical isolates. This figure shows the distribution of the best-match LS values for each of the 4 spots issued from identification of the 156 clinical isolates. The dark line indicates the best-match LS values of the concordant spots whereas the gray line shows the best-match LS values of the discordant ones. Concordant and discordant spots best-match LS value distributions were almost distinct.

For 150 (96.15%) of these 156 isolates, the MALDI-TOF MS-based species identification was correct. The intra-spot concordance was 100% for 141 of the 156 isolates i.e. the 4 spots yielded the same identification at the species level. Their corresponding best-match LS values were high. For the 9 remaining isolates, only three spots were concordant, whereas the fourth gave a discordant identification. As plotted in Figure 5, the mean best-match LS values were higher for the concordant spots (2.141±0.259) than for the discordant ones (1.341±0.189).

One isolate was misidentified as *Mucor circinelloides* by MALDI-TOF MS because the spectra of three concordant spots matched with *Mucor circinelloides* (mean best-match LS = 1.743±0.165), but the phenotypic/genotypic identification was *Rhizopus oryzae*.

In five isolates no MALDI-TOF MS-based identification was obtained because of intra-spot discordant results; each of the four spots matched with four distinct species' MSPs with low best-match LS values (mean = 1.224+/−0.195). A new subculture of these five isolates was subjected to a new extraction step, which markedly enhanced the spectra quality in four of these five isolates. Identification at the species level was correct for three isolates with four concordant spots with high best-match LS values (Table 2). Identification at the genus level was correct for one *Penicillium chrysogenum* isolate with three concordant spots (Table 2). The fifth isolate, identified as *Beauveria bassiana* by DNA sequence-based identification, could not be identified by MALDI-TOF MS because a new assay did not improve the previous results (the four spots were discordant with low best-match LS values) (Table 2).

**Table 2:** Best-match LS values of 5 isolates with intra-spot discordant results at the first identification assay.

	First MS Identification		Second MS Identification	
Isolate	Result	Score	Result	Score
***A. fumigatus***	*P. exophialae*	1. 301	*A. fumigatus*	2.009
	*A. fumigatus*	1. 460	*A. fumigatus*	2.114
	*A. niger*	1. 354	*A. fumigatus*	1.874
	*A. strictum*	1. 456	*A. fumigatus*	1.883
***S. apiospermum***	*S. inflatum*	1. 063	*S. apiospermum*	2.067
	*F. oxysporum*	0. 931	*S. apiospermum*	2.014
	*A. hollandicus*	0. 771	*S. apiospermum*	1.995
	*A. nidulans*	0. 994	*S. apiospermum*	1.903
***P. chrysogenum***	*Alternaria sp.*	0. 998	*P. roqueforti*	1.533
	*A. terreus*	1. 169	*P. roqueforti*	1.641
	*S. apiospermum*	1. 117	*P. roqueforti*	1.476
	*F. verticillioides*	1. 058	*P. aurantiogriseum*	1.636
***B. bassiana***	*B. bassiana*	1. 280	*M. circinelloides*	1.761
	*P. spirulosum*	1.308	*C. sphaerospermum*	1.760
	*Oedocephalum sp*	1.366	*M. circinelloides*	1.856
	*P. chermesinum*	1.226	*B. bassiana*	1.805
***A. niger***	*F. oxysporum*	1. 382	*A. niger*	2.021
	*A. nidulans*	1. 389	*A. niger*	2.174
	*P. chrysogenum*	1. 472	*A. niger*	1.960
	*F. oxysporum*	1. 381	*A. niger*	1.992

(MS: mass spectrometry).

In summary, from the clinical point of view, this MALDI-TOF MS-based approach was able to correctly identify 87% (154/177) of the isolates analyzed in a routine clinical laboratory activity. It failed in 12% (21/177), whose species were not represented in the reference library. When focusing on the 156 clinical isolates for which at least one MSP of the same species was present in the library, at least three concordant spots were obtained for 151 (96.8%) isolates, leading to an initial correct MALDI-TOF MS-based identification in 150 (96.15%) of them. When taking into account the results of a replicate analysis of all isolates that initially yielded less than three concordant spots, which is indicative of a possible technical error, the MALDI-TOF MS-based identification resulted in 154 (98.7%) correct identifications at the species level.

## Discussion

This is the first demonstration that a standardized MALDI-TOF procedure is capable to identify a large array of distinct mould species that are routinely isolated in the clinical laboratory setting. In this setting, MALDI-TOF MS-based identification has already revolutionized the identification of bacteria and yeasts [7]. A growing number of clinical laboratories are now equipped with MALDI-TOF MS-based solutions for the MALDI-TOF MS-based identification of micro-organisms. Yet the lack of standardized procedure applicable to the routine identification of moulds isolated in the clinical laboratory routine remained the major gap in commercialized solutions to date. It was thus critical to develop a similar solution for the identification of moulds.

Data on MALDI-TOF MS-based identification of moulds are scarce in the literature. The limited number of studies have focused on specific genera or phylogenetic complexes such as *Aspergillus*, *Penicillium*, *Trichophyton*, *Fusarium*, *Verticillium*, *Trichoderma*, and *Scedosporium* [14], [15], [16], [17], [18], [19], [20], [21], and each of them used heterogeneous fungal cultures or extraction procedures. For instance, the delay of culture varied from 48 h for De Respinis to 20 days for Hettick [14], [19], and extraction was performed from spores [9], [15], hyphae [18], [19], or both spores and hyphae [16], [17], [20], [22]. A wide array of extraction procedures have been used, including heating, sonication, bead-beating, or chemical lysis. DHB and α-HCCA matrix were mostly used but Welham et al. and Valentine et al. used a hydroxyphenylphenylbenzoic acid- and a ferrulic acid-based matrix, respectively [9], [22]. Here we selected an optimized procedure suited to the identification of the main relevant mould species in the clinical laboratory setting. Indeed, when challenging the subcultures of the strains included in our library, we obtained high best-match LS values, comparable to those obtained for bacteria or yeast identification [7].

As explained by Giebel et al., an ideal mass spectral identification system for moulds needs to be simple, with a high turnaround time, fast in handling, robust with respect to variations and variability in culture conditions, reproducible to allow identifications at different locations, applicable to the majority of clinically relevant microorganisms, and economical to allow identifications at competitive costs [23]. In our study, the first step resulted in an optimized extraction procedure adapted to the laboratory routine. We used Sabouraud-chloramphenicol-gentamicin agar, which is the most widely used fungal isolation medium in clinical laboratories. Few studies dealing with hyaline molds producing numerous conidia (*Aspergillus sp*. [22], [24], *Penicillium sp*. [15], *Rhizopus sp* [22]., *Trichoderma sp.* [22] and *Phanerochaete sp*. [22]) lead to reproducible mass fingerprints by intact fungal cells analysis. However we failed to obtain interpretable spectra from dematious, poorly- or non-sporulating molds. Thus we used a chemical extraction step to achieve a good quality of spectra from any molds. In keeping with Coulibaly et al. [21], formic acid-based extraction was chosen because it was faster, less toxic, and resulted in an identification quality similar to that of trifluoroacetic acid-based extraction.

An α-HCCA-based matrix was selected because it is extensively used for bacterial and yeast identification and because it had succeeded in identifying *Aspergillus*, *Fusarium*, and *Pseudallescheria/Scedosporium* isolates [7], [17], [18], [20], [25]. Except for those including a thermal lysis step, each tested extraction procedure yielded mass spectra profiles with >40 peaks. This clearly exceeds the 17-peak threshold ensuring the species specificity of a spectrum [16]. Finally procedure A, based on formic acid extraction, was selected because it was both the most reproducible and the easiest to perform in a routine setting.

The final evaluation of clinical isolates collected from the routine activity of our laboratory fairly succeeded. Testing the validity of our entire process in identifying clinical isolates in parallel with conventional methods, we identified 154 (87%) out of 177 isolates. These findings are very encouraging especially when considering that the reference library used was 20 times smaller than bacteria libraries. Our tentative library, which included only references for 146 strains belonging to only 63 species and 33 different genera, allowed the identification at the species level of 87% of the isolates identified in the routine activity of a clinical laboratory for 5 months. Seng et al. identified 84% of the bacterial isolates in clinical bacteriology laboratory routine with a 2881 reference library [7]. Since 2009 the MALDI-TOF MS based identification of bacteria increased with the number of reference spectra in the libraries. To date, our findings constitute a proof of concept that moulds identification can be adapted to the routine clinical laboratory and it is likely that MALDI-TOF MS based identification moulds will also benefit from strengthening reference libraries.

This novel standardized MALDI-TOF MS-based mould identification assay allowing the timely and accurate identification of clinically relevant moulds at the species level in the routine microbiology laboratory setting is likely to dramatically alter the management of fungal infections. Our findings demonstrate that MALDI-TOF MS identification is efficient for the rapid and routine identification of mould isolates in the clinical laboratory and, in line with the current practices of bacteria identification in the growing number of microbiology laboratories equipped with bench-top MALDI-TOF instruments, it could be used, ahead of morphological identification, as a first-line method for mould identification. Additionally, this MALDI-TOF MS identification process will have a great impact on several other research areas that would benefit from a high throughput and accurate mould identification assay. Indeed, moulds are of growing interest in human health, food safety management, and the control of phytopathogenic fungi. Some species (i.e. in *Penicillium* or *Aspergillus* genera) have been associated with allergic diseases [26]. In contrast, Ege et al. recently pointed to the significant protection against childhood asthma associated with exposure to farm microbiota, and especially fungal taxa [27]. Furthermore, the human-health consequences of mycotoxins produced by species of *Aspergillus*, *Penicillium*, *Claviceps*, *Fusarium*, and *Alternaria* are of concern, as is the burden of phytopathogenic fungi on farming [28]. With its potential to identify a wide array of microorganism species at the strain level within minutes, this unique microorganism identification approach will indubitably increase our understanding of the complex human health and environmental microbiota interactions in the very near future.

MALDI-TOF MS recently became one of the routine microorganism identification tools in the clinical laboratory. This work's seminal finding is that, akin to bacteria and yeasts, a standardized procedure can also be used for MALDI-TOF MS-based identification of a wide array of clinically relevant mould species. Usable in the routine clinical laboratory setting, it opens new avenues for the development of an integrated MALDI-TOF MS-based solution for the identification of any clinically relevant microorganisms.

## Supporting Information

Table S1 **Fungal strains of this study by source and the panel in which they were included.** (Nb.: number; IHEM: Institut d'Hygiene et d'Epidemiologie, Section de Mycologie, Brussels, Belgium; CS : clinical strain ; ES : environmental strain ; PI : Pasteur Institute; P1: panel 1; P2: Panel 2; P3: Panel 3).
(DOCX)
