# Supplementary material for: Mould Routine Identification in the Clinical Laboratory by Matrix-Assisted Laser Desorption Ionization Time-Of-Flight Mass Spectrometry
Source: PLoS One. 2011 Dec 14;6(12):e28425. doi: 10.1371/journal.pone.0028425 (PMC3237453; doi:10.1371/journal.pone.0028425)
Supplement: Table S1 — Fungal strains of this study by source and the panel in which they were included. (Nb.: number; IHEM: Institut d'Hygiene et d'Epidemiologie, Section de Mycologie, Brussels, Belgium; CS : clinical strain ; ES : environmental strain ; PI : Pasteur Institute; P1: panel 1; P2: Panel 2; P3: Panel 3). (DOCX) [file pone.0028425.s001.docx]

| **Species** | **Strains nbr** | **Strain name** | **Source** | **In panel** |
| --- | --- | --- | --- | --- |
| *Absidia coerula* | 1 | Pasteur 181 | PI | P3 |
| *Absidia (Lichtheimia) corymbifera* | 4 | IHEM16288 | IHEM | P3 |
|  |  | IHEM14734 | IHEM | P3 |
|  |  | IHEM21658 | IHEM | P3 |
|  |  | ABSI001 | CS | P2, P3 |
| *Acremonium strictum* | 3 | Pasteur 173 | PI | P3 |
|  |  | IHEM19179 | IHEM | P3 |
|  |  | IHEM 22371 | IHEM | P3 |
| *Acrophialophora fusispora* | 3 | IHEM15939 | IHEM | P3 |
|  |  | IHEM19591 | IHEM | P3 |
|  |  | IHEM19730 | IHEM | P3 |
| *Alternaria alternata* | 6 | ALTE004 | CS | P1, P2, P3 |
|  |  | ALTE006 | CS | P2, P3 |
|  |  | Pasteur 120 | PI | P3 |
|  |  | IHEM22669 | IHEM | P3 |
|  |  | IHEM21999 | IHEM | P3 |
|  |  | IHEM9788 | IHEM | P3 |
| *Alternaria tenuissima* | 1 | Pasteur 121 | PI | P3 |
| *Aspergillus candidus* | 4 | Pasteur 114 | PI | P3 |
|  |  | IHEM15975 | IHEM | P3 |
|  |  | IHEM14607 | IHEM | P3 |
|  |  | IHEM9678 | IHEM | P3 |
| *Aspergillus flavus* | 5 | IHEM23376 | IHEM | P3 |
|  |  | IHEM14475 | IHEM | P3 |
|  |  | AFLA002 | CS | P1, P2, P3 |
|  |  | 1006130 | CS | P3 |
|  |  | 1027804 | CS | P3 |
| *Aspergillus fumigatus* | 10 | IHEM15161 | IHEM | P3 |
|  |  | IHEM19416 | IHEM | P3 |
|  |  | IHEM22145 | IHEM | P3 |
|  |  | AFUM001 | CS | P1, P2, P3 |
|  |  | AFUM002 | CS | P2, P3 |
|  |  | 1030590 | CS | P3 |
|  |  | IHEM22112 | IHEM | P3 |
|  |  | 1008277 | CS | P3 |
|  |  | 1005559 | CS | P3 |
|  |  | 1007844 | CS | P3 |
|  |  | Pasteur 122 | PI | P3 |
| *Aspergillus hollandicus* | 1 | ST55 | ES | P3 |
| *Aspergillus lentulus* | 1 | IHEM22112 | IHEM | P3 |
| *Aspergillus nidulans* | 5 | IHEM23179 | IHEM | P3 |
|  |  | IHEM23366 | IHEM | P3 |
|  |  | ST41 | ES | P3 |
|  |  | ST42 | ES | P3 |
|  |  | Pasteur 123 | PI | P3 |
| *Aspergillus nigri section* | 4 | IHEM18551 | IHEM | P3 |
|  |  | IHEM9673 | IHEM | P3 |
|  |  | IHEM5077 | IHEM | P3 |
|  |  | ANIG001 | CS | P1, P2, P3 |
| *Aspergillus ochraceus* | 1 | Pasteur 115 | PI | P3 |
| *Aspergillus sydowi* | 1 | CQB436 | IHEM | P3 |
| *Aspergillus terreus* | 7 | IHEM17777 | IHEM | P3 |
|  |  | IHEM18939 | IHEM | P3 |
|  |  | IHEM9995 | IHEM | P3 |
|  |  | ST46 | ES | P3 |
|  |  | ATER002 | ES | P1, P2, P3 |
|  |  | ATER003 | ES | P2, P3 |
|  |  | ATER004 | ES | P2, P3 |
| *Aspergillus ustus* | 2 | IHEM20348 | IHEM | P3 |
|  |  | IHEM16237 | IHEM | P3 |
| *Aspergillus versicolor* | 2 | IHEM22074 | IHEM | P3 |
|  |  | Pasteur 124 | IHEM | P3 |
| *Aureobasidium pullulans var. lata* | 1 | IHEM18870 | IHEM | P3 |
| *Beauveria bassiana* | 4 | IHEM6954 | IHEM | P3 |
|  |  | Pasteur 146 | PI | P3 |
|  |  | BEAU001 | CS | P2, P3 |
|  |  | IHEM18747 | IHEM | P3 |
| *Chaetonium globosum* | 1 | CQB684 | IHEM | P3 |
| *Chrysonilia sp.* | 1 | ST119 | ES | P3 |
| *Cladosporium carionii* | 1 | Pasteur 128 | PI | P3 |
| *Cladosporium cladosporioides* | 1 | IHEM9072 | IHEM | P3 |
| *Cladosporium herbarum* | 1 | IHEM14610 | IHEM | P3 |
| *Cladosporium sphaeropermum* | 1 | IHEM17164 | IHEM | P3 |
| *Curvularia sp.* | 1 | Pasteur 131 | PI | P3 |
| *Emericiella nidulans* | 2 | Pasteur 130 | PI | P3 |
|  |  | IHEM19096 | IHEM | P3 |
| *Eurotium amstelodami* | 1 | Pasteur 175 | PI | P3 |
| *Eurotium chevalieri* | 1 | CQB103.4 | IHEM | P3 |
| *Exophiala dermatitidis* | 3 | EXOP001 | CS | P1, P2, P3 |
|  |  | IHEM23421 | IHEM | P3 |
|  |  | IHEM9780 | IHEM | P3 |
| *Exophiala phaeomuriformis* | 1 | IHEM20746 | IHEM | P3 |
| *Fusarium equiseti* | 1 | ST132 | ES | P3 |
| *Fusarium oxysporum* | 4 | IHEM18448 | IHEM | P3 |
|  |  | FUSA003 | CS | P1, P2, P3 |
|  |  | ST62 | CS | P3 |
|  |  | ST73 | CS | P3 |
| *Fusarium solani* | 3 | IHEM22015 | IHEM | P3 |
|  |  | IHEM7504 | IHEM | P3 |
|  |  | 1000694 | CS | P3 |
| *Fusarium verticillioides* | 3 | IHEM20180 | IHEM | P3 |
|  |  | IHEM22962 | IHEM | P3 |
|  |  | IHEM18495 | IHEM | P3 |
| *Geomyces pannorum* | 1 | IHEM2667 | IHEM | P3 |
| *Humicola sp.* | 1 | Pasteur 132 | PI | P3 |
| *Irpex lacteus* | 1 | ST32 | CS | P3 |
| *Mucor circinelloides* | 1 | ST45 | ES | P3 |
| *Neosartorya pseudoficheri* | 2 | IHEM23148 | IHEM | P3 |
|  |  | IHEM23044 | IHEM | P3 |
| *Oedocephalum sp.* | 1 | Pasteur 177 | PI | P3 |
| *Paecilomyces variotii* | 4 | IHEM17703 | IHEM | P3 |
|  |  | IHEM3285 | IHEM | P3 |
|  |  | IHEM16627 | IHEM | P3 |
|  |  | Pasteur 127 | PI | P3 |
| *Penicillium lilacinus* | 1 | RHIZ002 | CS | P2, P3 |
| *Penicillium aurantiogriseum* | 3 | IHEM18723 | IHEM | P3 |
|  |  | IHEM20176 | IHEM | P3 |
|  |  | IHEM20357 | IHEM | P3 |
| *Penicillium brevicompactum* | 1 | IHEM22668 | IHEM | P3 |
| *Penicillium chermesinum* | 1 | PENI007 | CS | P2, P3 |
| *Penicillium chrysogenum* | 6 | IHEM17894 | IHEM | P3 |
|  |  | IHEM22667 | IHEM | P3 |
|  |  | CQB435 | IHEM | P3 |
|  |  | IHEM20859 | IHEM | P3 |
|  |  | Pasteur 118 | PI | P3 |
|  |  | PENI006 | CS | P2, P3 |
| *Penicillium corylophylus* | 1 | Pasteur 125 | PI | P3 |
| *Penicillium purpurogenum* | 1 | Pasteur 116 | PI | P3 |
| *Penicillium roqueforti* | 1 | Pasteur 119 | PI | P3 |
| *Penicillium spinulosum* | 1 | Pasteur 117 | PI | P3 |
| *Phialophora parasitica* | 1 | Pasteur 139 | PI | P3 |
| *Pseudallescheria boydii* | 1 | IHEM14263 | IHEM | P2, P3 |
| *Rhizomucor pusillus* | 3 | IHEM21236 | IHEM | P3 |
|  |  | IHEM18686 | IHEM | P3 |
|  |  | IHEM16462 | IHEM | P3 |
| *Rhizopus oryzae* | 4 | IHEM13186 | IHEM | P3 |
|  |  | IHEM21660 | IHEM | P3 |
|  |  | MUCO002 | CS | P2, P3 |
|  |  | IHEM16287 | IHEM | P3 |
| *Scedosporium apiospermum* | 5 | IHEM3817 | IHEM | P3 |
|  |  | IHEM6908 | IHEM | P3 |
|  |  | IHEM14632 | IHEM | P3 |
|  |  | SFMM1 | CS | P3 |
|  |  | Pasteur 174 | PI | P3 |
| *Scedosporium prolificans* | 3 | IHEM5739 | IHEM | P3 |
|  |  | IHEM18755 | IHEM | P3 |
|  |  | IHEM22339 | IHEM | P3 |
| *Schizophyllum commune* | 1 | ST33 | CS | P3 |
| *Scopulariopsis brevicaulis* | 3 | IHEM15574 | IHEM | P3 |
|  |  | IHEM1690 | IHEM | P3 |
|  |  | IHEM22982 | IHEM | P3 |
| *Trichoderma viride* | 2 | TRIC001 | ES | P1, P2, P3 |
|  |  | IHEM3170 | IHEM | P3 |
| *Trichotecium roseum* | 3 | IHEM1535 | IHEM | P3 |
|  |  | IHEM7941 | IHEM | P3 |
|  |  | IHEM2478 | IHEM | P3 |
| *Ulocladium sp.* | 1 | Pasteur 133 | PI | P3 |
